# Genomic insights into Mediterranean pepper diversity using ddRADSeq

**DOI:** 10.1371/journal.pone.0318105

**Published:** 2025-03-10

**Authors:** Tuğba Pelin Toker, Damla Ulusoy, Betül Doğan, Serkan Kasapoğlu, Fidan Hakan, Umesh K. Reddy, Mojtaba Kordrostami, Engin Yol

**Affiliations:** 1 Department of Field Crops, Faculty of Agriculture, Akdeniz University, Antalya, Turkiye; 2 Anamas Tarım Ltd. Şti. Antalya, Turkiye; 3 Department of Plant Protection, Faculty of Agriculture, Akdeniz University, Antalya, Turkiye; 4 Department of Biology, Gus R. Douglass Institute, West Virginia State University, Institute, West Virginia, United States of America; 5 Nuclear Agriculture Research School, Nuclear Science and Technology Research Institute (NSTRI), Karaj, Iran; National Cheng Kung University, TAIWAN

## Abstract

This work investigated the genetic diversity and population structure of 99 pepper lines (*Capsicum annuum* L.), acclimated to Mediterranean climate conditions, using double-digest restriction site-associated DNA sequencing (ddRADSeq). The aims were to understand the genetic relationships among these lines, correlate genetic clusters with botanical classifications, and provide insights into pepper domestication in the region. Obtained were 318.76 million raw sequence reads overall, averaging 3.21 million reads per sample. A total of 8475 high-quality SNPs were identified and used to assess genetic diversity and population structure. Chromosome NC_061113.1 displayed the highest amount and Chromosome NC_061118.1 the fewest of these SNPs, which were not equally spaced around the genome. Heterozygosity measures and a negative inbreeding coefficient point to the great genetic diversity seen, therefore highlighting the genetic health of the population. Different genetic clusters found by phylogenetic study and STRUCTURE analysis can be used in breeding programs to mix desired features from many genetic backgrounds. This work showed how well ddRADSeq generates high-quality SNPs for genomic research on peppers, therefore offering useful molecular tools for genomic selection and marker-assisted selection. The analysis identified significant genetic diversity and distinct genetic clusters which are valuable for breeding programs focused on crop improvement. These findings enhance our understanding of pepper domestication and provide valuable genetic resources for breeding programs aimed at improving pepper varieties.

## 1. Introduction

Plant domestication has been a pivotal catalyst of human civilization, converting wild species into crops that have supported society for millennia [1,2]. This process, initiated some 12,000 years ago, entailed the selection of plants exhibiting favorable characteristics such as enhanced production, palatability, and cultivational simplicity. Domestication has induced substantial morphological, physiological, and genetic alterations in plants, culminating in the varied spectrum of crops produced today [3,4].

Peppers (*Capsicum* spp.) are significant among these crops due to their economic relevance, nutritional benefits, and cultural importance [5,6]. The genus *Capsicum* comprises more than 35 species, with five domesticated species identified: *Capsicum annuum*, *C. chinense*, *C. frutescens*, *C. baccatum*, and *C. pubescens* [7]. Peppers originated in the Americas and have been farmed for at least 6,000 years, rendering them one of the first domesticated plants in the New World [8].

The domestication of peppers occurred through several discrete stages in various locations of the Americas. Archaeological evidence indicates that peppers were initially domesticated in areas currently including Mexico and Bolivia [9,10]. Domestication resulted in substantial modifications to fruit attributes, encompassing enhanced size, diverse shapes and colors, and changes in capsaicinoid levels, which influence pungency. These characteristics were chosen according to human preferences for culinary applications, medical uses, and cultural traditions [11].

Pepper domestication has been pivotal in shaping its genetic diversity and phenotypic traits, both of which are vital for breeding efforts and agricultural sustainability. Recent genomic studies have provided usefull insights into the domestication processes and genetic architecture of *Capsicum* species. For instance, Liu et al. [12] uncovered the complex domestication history and gene flow events among wild and cultivated peppers, highlighting the genetic basis of key domestication traits [12]. Moreover, Cao et al. [13] identified key genomic regions associated with fruit size, shape, and capsaicinoid content, which are vital for understanding the domestication syndrome in peppers. Their work emphasized the role of human selection in shaping these traits during domestication [13]. Additionally, Kim et al. [14] demonstrated the impact of domestication on the genomic diversity of *Capsicum annuum*, revealing selective sweeps and genetic bottlenecks that occurred during the transition from wild to cultivated forms [14].

Understanding the genetic diversity and domestication history of peppers is crucial for advancing crop improvement and conservation efforts. Domestication frequently leads to genetic bottlenecks, diminishing the genetic diversity within cultivated species relative to their wild counterparts [15]. Wild *Capsicum* species serve as significant reservoirs of genetic variety, containing alleles for disease resistance, abiotic stress tolerance, and other agronomically relevant traits [16,17]. Progress in genetic technologies has yielded novel insights about the domestication and evolution of peppers. Whole-genome sequencing and resequencing investigations have pinpointed genetic loci linked to domestication characteristics and adaptation [18,19]. These studies have revealed the genetic basis of key domestication traits and the complex evolutionary history of peppers.

Population genetic investigations have clarified the connections between cultivated peppers and their wild progenitors. Observations indicate gene flow between cultivated and wild populations, suggesting that introgression has contributed to the evolution of pepper types [20,21]. Comprehending these dynamics is crucial for preserving genetic resources and employing wild material in breeding initiatives [22].

In the Mediterranean region, peppers are fundamental to agriculture and food, with various regionally adapted varieties that display distinct morphological and sensory characteristics [23]. Varieties like Dolma, Sivri, Kapia, and Charleston peppers hold significant importance in local cuisines and cultural traditions [24]. Notwithstanding their significance, there is an absence of thorough genetic research on Mediterranean-adapted pepper lines [25]. Understanding the genetic variety of these types is vital for breeding efforts focused on enhancing stress tolerance and production, given the challenges of climate change and the necessity for sustainable agriculture [26].

Despite the advances in molecular sciences for studying pepper, there is a lack of comprehensive studies focusing on pepper lines adapted to Mediterranean climates, which are characterized by unique environmental stresses [27]. Our study aimed to fill this gap by analyzing the genetic diversity and population structure of 99 Mediterranean-adapted pepper lines using ddRADSeq, providing valuable insights for targeted breeding programs. The specific objectives of this study were: a) to characterize the genetic diversity of 99 Mediterranean-adapted pepper promising lines using ddRADSeq generated SNP markers. b) to determine the population structure of these pepper lines and identify genetic clusters. c) to identify private alleles and SNP markers associated with specific pepper types, which can be used in marker-assisted breeding programs.

## 2. Materials and methods

### 2.1 Plant materials

The 99 pepper lines (*Capsicum annuum* L.) were collected from diverse agricultural regions across Turkey, representing the genetic variability present in Mediterranean-adapted peppers (S1 Table). Selection criteria included variation in fruit morphology, taste (sweet or hot), and traditional use. Pepper lines were classified into types (e.g., Dolma, Sivri, Charleston) based on standardized morphological descriptors such as fruit shape, size, color, and pungency levels, following the guidelines of the International Union for the Protection of New Varieties of Plants (UPOV) [28]. Our sampling design was structured to capture the genetic diversity of Mediterranean-adapted pepper varieties. While initial sampling plans considered the inclusion of both cultivated and wild (undomesticated) peppers, we ultimately did not include any truly wild accessions. All 99 lines represent cultivated varieties or landraces adapted to Mediterranean conditions, sourced from genebanks and breeding programs. This set includes traditional landraces that retain some ancestral traits but does not encompass any undomesticated wild *Capsicum* populations. We selected 99 pepper lines representing different pepper types (e.g., Charleston, Kil, Sivri, etc.) based on their adaptation to Mediterranean climatic conditions, morphological diversity, and availability in genebanks and breeding programs. The sampling aimed to include sufficient genetic variation within each type to allow robust comparisons in our population genetic analyses. Additionally, we ensured that all major domesticated groups and local landraces were represented to assess both historical domestication and modern breeding impacts. The number of lines for each variety was chosen to balance representation while managing practical constraints such as sample processing and sequencing capacity.

To determine the variety to which each sample belongs, we utilized key morphological characteristics that are commonly used for classifying pepper varieties (S1 Table). Specifically, we relied on traits such as fruit shape (blocky, conical, elongated), fruit size, color, pungency level, and plant habit (growth form, branching pattern). These characteristics were assessed based on established botanical keys and descriptors from published literature on *Capsicum* varieties. In addition, information from the genebanks and local breeding programs where these samples were sourced helped confirm the identification. These morphological features allowed us to reliably assign each sample to its respective variety, ensuring consistency across our dataset.

### Botanical classification and origin

- **Sivri (25 lines):**
  ✓ **Botanical Classification:** *C. annuum* var. *longum*
  ✓ **Origin:** Predominantly Turkey
  ✓ **Description:** Long, thin fruits; green to red when ripe; range from mild to very hot.
  ✓ **Distribution:** Widely cultivated in Turkey and neighboring Mediterranean regions.
- **Dolma (21 lines):**
  ✓ **Botanical Classification:** *C. annuum* var. *grossum*
  ✓ **Origin:** Turkey and Mediterranean countries
  ✓ **Description:** Large, blocky fruits suitable for stuffing; sweet flavor.
  ✓ **Distribution:** Common in Mediterranean cuisine for dishes like stuffed peppers.
- **Kil (12 lines):**
  ✓ **Botanical Classification:** *C. annuum* var. *longum*
  ✓ **Origin:** Turkey
  ✓ **Description:** Long, curly fruits; can be sweet or hot.
  ✓ **Distribution:** Grown in Mediterranean regions.
- **Charleston (19 lines):**
  ✓ **Botanical Classification:** *C. annuum* var. *longum*
  ✓ **Origin:** Turkey
  ✓ **Description:** Horn-shaped peppers; soft flesh; fruity flavor.
  ✓ **Distribution:** Cultivated in Turkey and nearby areas.
- **Kapia (12 lines):**
  ✓ **Botanical Classification:** *C. annuum* var. *grossum*
  ✓ **Origin:** Mediterranean region
  ✓ **Description:** Small, tapered fruits; vivid red color; sweet taste.
  ✓ **Distribution:** Popular in Mediterranean cuisines.
- **Mazamort (4 lines):**
  ✓ **Botanical Classification:** *C. annuum* var. *grossum*
  ✓ **Origin:** Specific regions in Turkey
  ✓ **Description:** Unique triple-pointed fruits; crisp and sweet.
  ✓ **Distribution:** Localized cultivation.
- **Chili (4 lines):**
  ✓ **Botanical Classification:** *C. annuum* var. *annuum*
  ✓ **Origin:** Various
  ✓ **Description:** Small, very hot peppers.
  ✓ **Distribution:** Grown worldwide; included for diversity.
- **Jalapeño (2 lines):**
  ✓ **Botanical Classification:** *C. annuum* var. *annuum*
  ✓ **Origin:** Mexico
  ✓ **Description:** Medium-sized, pungent fruits; dark green to red when ripe.
  ✓ **Distribution:** Cultivated globally; included to represent hot pepper diversity.

Phenotypic traits, including taste (sweet or chili) and fruit color, were recorded for each pepper line. These traits are crucial for linking genetic markers to agronomic characteristics of interest. By correlating phenotypic variation with genetic data, we aimed to identify SNPs linked to specific traits, facilitating their use in breeding programs.

### 2.2 DNA extraction and ddRAD sequencing

DNA was isolated from fresh leaves using a modified CTAB protocol [29], with a few minor modifications, including the addition of extra chloroform-isoamyl alcohol and additional cleaning stages using 70% ethanol. The DNA quantity and quality were evaluated by running it on a 1% agarose gel.

ddRADSeq libraries were prepared by digesting the genomic DNA with the restriction enzymes *Vsp*I and *Msp*I, followed by ligation of barcoded adapters specific to each sample. After pooling the samples, size selection was performed to remove unligated adapters and small fragments. The libraries were then enriched by PCR and subjected to Illumina 150 bp paired-end (PE) sequencing on the HiSeq platform. Libraries were prepared following the protocol published by Peterson et al. [30], with minor modifications. Unlike the original protocol, we selected the six-base cutter *Vsp*I restriction enzyme instead of *Eco*RI. High-throughput sequencing was performed on an Illumina HiSeq 4000 platform. The ddRADSeq data have been deposited in the National Center for Biotechnology Information (NCBI) Sequence-Read Archive (SRA) database with the accession number of PRJNA1129584.

### 2.3 SNP calling

The raw data were demultiplexed using Je (v1.2) [31] and arranged into distinct FASTQ files specific to each genotype. Fastp [32] was used with default parameters for a quality check. After the filtering process, each individual FASTQ file was aligned to the *Capsicum annuum* reference genome “UCD10Xv1.1” [19] using Bowtie2 software [33] with default parameters. The Galaxy software framework (www.usegalaxy.org) was utilized to run the SNP calling tools.

In the first step of SNP calling, genotype-specific individual BAM (binary sequence alignment) files were analyzed with FreeBayes (Galaxy Version 1.1.0.46–0) [34] to identify variants, with the parameters set to simple diploid calling and coverage values set to 20X. Only SNPs were retained; insertions and deletions (InDels) were removed from each VCF file using VCFfilter (Galaxy Version 1.0.0). A minimum allele frequency of 0.05 was used to exclude rare alleles that may represent sequencing errors, ensuring robustness in our genetic diversity estimates [35]. Following the filtering step, genotype-specific VCF files were merged using VCFgenotypes (Galaxy Version 1.0.0) to form a single data file. Finally, the merged SNPs were filtered using Tassel V5.2.52 [36] with the following parameters: a site minimum count of 50 to ensure sufficient representation across samples, a Minor Allele Frequency (MAF) threshold of >  0.05 to retain polymorphic loci, and the exclusion of sites containing indels to ensure accuracy in population genetics analysis. This choice ensures that only alleles present at a moderate frequency across the population are retained, thereby reducing the risk of including spurious variants resulting from sequencing errors or extremely low coverage [37,38]. We acknowledge that this MAF cutoff may lead to the exclusion of rare alleles, potentially including those unique to varieties represented by very few individuals. To assess the impact of MAF filtering on our dataset, we tested alternative thresholds (e.g., 0.01 and 0.02) prior to final selection. While lower MAF thresholds slightly increased the number of rare alleles, it also introduced more potential errors and decreased overall data quality [39,40]. Thus, we have chosen a MAF of 0.05 as a balance between data robustness and the retention of potentially informative alleles. We recognize that this filtering may have reduced the detection of unique private alleles in small groups, and this limitation is considered when interpreting the results. Private alleles were identified by calculating allele frequencies across different pepper types [41]. Alleles with a frequency greater than zero in one type and zero in all others were considered private [41].

After quality filtering and SNP calling, we assessed missing data and sequencing depth for each of the 99 pepper lines. The proportion of missing genotype calls per individual was calculated as the number of missing genotypes divided by the total number of genotypes scored. Sequencing depth was calculated as the average number of reads per SNP per individual. On average, each line showed 4.5% missing data (ranging from 2.3% to 7.2%) and a mean sequencing depth of approximately 18 × (ranging from 15 × to 22×).

### 2.4 Diversity analyses

#### 2.4.1. Genetic diversity metrics.

Population genetics statistics, including measures of genetic diversity such as observed heterozygosity (Ho), expected heterozygosity (He), the number of alleles (Na), and genetic differentiation (FST), were calculated using GenAlex V6.5 [42]. These statistics were used to assess the genetic variability within and between pepper lines.

**2.4.2. Population structure (STRUCTURE analysis):** The population structure was determined using the Bayesian clustering approach implemented in STRUCTURE V2.3.4 [43], employing the admixture model based on allele frequencies. A burn-in period of 10,000 Markov Chain Monte Carlo (MCMC) iterations and a run length of 100,000 were used to identify the number of populations (K) present within the 99 genotypes. These parameters were chosen based on their reliability in achieving convergence and ensuring the accuracy of population structure analysis in similar studies [44–46]. Ten independent runs were performed for each simulated value of K, ranging from 2 to 10. The optimal K was determined using Structure Harvester [47] (http://taylor0.biology.ucla.edu/structureHarvester/). Each pepper genotype was then assigned to a cluster (Q) based on probability. The population structure bar plot was generated using the STRUCTUREPLOT V2.0 web-based tool [48] (http://omicsspeaks.com/strplot2/), ordered by Q-value.

**2.4.3. Principal coordinate analysis (PCoA) and phylogenetic tree:** Principal coordinate analysis (PCoA) was conducted with PAST V3.23 [49]. A phylogenetic tree was constructed using the unweighted pair group method with arithmetic mean (UPGMA) [50] in Tassel V5.2.52 and modified in FigTree V1.4.4 (http://tree.bio.ed.ac.uk/software/figtree). The UPGMA method was selected for its ability to cluster genetic distances between genotypes, providing an efficient means of visualizing relationships among closely related pepper lines, despite its assumption of equal evolutionary rates across lineages [51].

**2.4.4. Inbreeding (runs of homozygosity- Froh):** To assess the level of inbreeding and identify long stretches of homozygous regions across the genome, the runs of homozygosity (Froh) was calculated for each individual within the pepper population [52]. Froh is defined as the proportion of the genome that is homozygous in relation to the total genome length, providing a more precise estimate of inbreeding by accounting for long homozygous segments that may have arisen through recent common ancestry [53]. The Froh values were computed using the PLINK v1.9 software, which identifies continuous homozygous segments (runs) of single nucleotide polymorphisms (SNPs) within the genome. The following parameters were used to define a run of homozygosity [54]: a minimum run length of 500 kb, a maximum gap between consecutive SNPs of 50 kb, and at least 50 SNPs per run. Additionally, no more than one heterozygous site and no more than five missing SNPs were allowed within a single run to ensure the accuracy of detected homozygous regions. The resulting Froh values were calculated as the proportion of the genome covered by homozygous regions relative to the total number of SNPs analyzed for each individual [55]. These values were then averaged across all individuals within the population to obtain a mean Froh, which was used to assess the degree of inbreeding across the different pepper varieties.

**2.4.5. Nucleotide diversity and linkage disequilibrium (LD) decay**: Nucleotide diversity (π) and linkage disequilibrium (LD) decay were calculated to assess genetic variation and recombination rates within the pepper population [56,57]. Nucleotide diversity, representing the average number of nucleotide differences per site between any two randomly chosen sequences, was calculated using VCFtools with the command vcftools --vcf input_data.vcf --site-pi --out nucleotide_diversity, and the values were averaged across the genome and for each pepper line [58]. Linkage disequilibrium (LD) decay, which describes how the correlation (r²) between pairs of SNPs decreases with increasing genetic distance, was analyzed using PLINK [59]. The VCF file was first converted to PLINK format (plink --vcf input_data.vcf --make-bed --out pepper_data), and pairwise LD was calculated with the command plink --bfile pepper_data --r2 --ld-window-kb 1000 --ld-window 99999 --ld-window-r2 0 --out ld_decay. The resulting LD decay plot was generated by plotting the r² values against the genetic distance between SNP pairs using Python’s matplotlib library [59].

**2.4.6. Transitions and transversions analysis:** The analysis of transitions and transversions was conducted using VCFtools v0.1.16. The filtered SNP dataset was analyzed to determine the number of transition (Ti) and transversion (Tv) mutations. The ratio of transitions to transversions (Ti/Tv ratio) was calculated to provide insight into the mutational patterns present in the pepper germplasm. This information is important as it helps to evaluate the quality of the SNP dataset and to understand the evolutionary processes shaping genetic variation in the population.

## 3. Results

### 3.1 Genotyping

After quality filtering, a total of 318.76 million filtered sequence reads were obtained from 150 bp paired-end sequencing on the Illumina HiSeq platform (S1 Table). Following the bioinformatic pipeline, 8475 filtered polymorphic SNP markers were identified and used for population genetic analysis. The mean number of reads per sample was 3.21 million, with an average guanine-cytosine (GC) content of 37.66%. Notably, the genotype ANS121, which belongs to the Kapia type, had the highest number of reads, reaching 5.21 million after filtering (S1 Table).

A total of 8475 high-quality SNPs were identified and used in the genetic diversity and population structure analyses. Out of these, 3495 SNPs were located on scaffolds, and the remaining 4980 SNPs were mapped to chromosomes (Table 1). All analyses were performed using the complete set of 8475 SNPs to ensure comprehensive genome-wide coverage. At the chromosomal level, Chromosome NC_061113.1 exhibited the highest number of SNPs, with 645 markers, while Chromosome NC_061118.1 had the fewest, with only 317 SNPs. The mean SNP density across the genome was 526.69 kb, with Chromosome NC_061115.1 showing the highest density at 621 SNP/kb and Chromosome NC_061112.1 displaying the lowest density at 412 SNP/kb (Table 1).

**Table 1:** Distribution SNPs in of each chromosome.

Chromosome	Initial number of SNPs	The filtered number of SNPs	Average map length per filtered SNP (kb)
Entire genome	4120941	8475	315
NC_061111.1	374481	490	522
NC_061112.1	273688	375	412
NC_061113.1	418134	645	420
NC_061114.1	306678	374	619
NC_061115.1	258345	356	621
NC_061116.1	312253	425	537
NC_061117.1	276882	388	586
NC_061118.1	267237	317	548
NC_061119.1	257904	415	528
NC_061120.1	273478	359	618
NC_061121.1	289064	421	560
NC_061122.1	289356	415	561
Scaffold	523441	3495	–

The observed SNPs exhibited different frequencies, with transitions and transversions identified and categorized using VCFtools. These frequencies were influenced by various evolutionary forces, including mutation, natural selection, genetic drift, and gene flow, shaping the genetic diversity across the pepper lines. The most frequent SNPs were C transitions, followed by G transitions. These transitions were the most common due to the higher mutation rates associated with them. For example, the deamination of cytosine can lead to uracil, which is then replaced by thymine, resulting in a C to T transition. Similarly, the deamination of adenine can lead to hypoxanthine, which is replaced by guanine, resulting in a G to A transition. In contrast, transversions, which involve purine-to-pyrimidine changes (such as A and G), were less frequent due to the larger molecular changes required and the structural constraints in DNA. Specifically, C transitions accounted for the highest proportion of SNPs at 16.53%, followed closely by G transitions at 15.80%. A and T transitions were also common, representing 14.53% and 14.17% of the SNPs, respectively. On the other hand, the least frequent SNPs were G transversions, making up only 2.53% of the SNPs, and C transversions, which constituted 2.89% (Table 2).

**Table 2:** SNP statistics in filtered data indicating number of allele and frequency of allele occurrence.

Allele*	Number of allele	Proportion of allele occurrence
C:T	1401	16.53
G:A	1339	15.80
A:G	1231	14.53
T:C	1201	14.17
T:A	498	5.88
A:T	491	5.79
A:C	486	5.73
C:A	471	5.56
G:T	460	5.43
T:G	438	5.17
C:G	245	2.89
G:C	214	2.53

*The first and second letters are reference and alternate alleles.

Evaluation of data quality across the 99 pepper lines revealed a relatively low proportion of missing genotypes and sufficient sequencing depth for reliable SNP calling (S1 Table). The average missing data per individual was 4.5%, with the best-covered samples (e.g., ANS005 and ANS032) exhibiting as little as ~ 2.5% missing data, and the most affected samples (e.g., ANS090) reaching up to ~ 7.0%. Likewise, the average sequencing depth per SNP per individual was approximately 18 × , ensuring confidence in genotype calls and minimizing the likelihood of false variants due to low coverage. This level of data completeness and coverage provides a robust foundation for the accurate estimation of genetic diversity, population structure, and related parameters

Private alleles are alleles unique to a single population or group and absent in others. In this study, private alleles were identified by screening the SNP dataset for alleles present exclusively in one pepper type and not detected in any other types. However, the low number of samples for some varieties may have influenced the detection and estimation of private alleles, potentially underrepresenting the true genetic variation within these varieties [60]. On Chromosome 1, private alleles were found in Charleston, Chili, Kil, and Jalapeño types with the specific alleles being A for Charleston and G for Chili, Kil, and Jalapeño (Table 3). On Chromosome 5, Kil and Mazamort types exhibited private alleles, represented by Y (C) for Kil and A for Mazamort. Chromosome 2 had private alleles in Kil, Jalapeño, and Sivri types, with the alleles being T for Kil and Sivri, and G for Jalapeño. Additionally, private alleles were identified on scaffolds SNW-025845820.1 and SNW-025893768.1 in Kil and Mazamort types, where the alleles were C and T for SNW-025845820.1, and C and W (A) for SNW-025893768.1.

**Table 3:** Private alleles in different pepper line groups.

Chromosome	Position	Allele-Charleston	Allele-Chili	Allele-Kil	Allele-Jalapeño	Allele-Mazamort	Allele-Sivri
Chr1	1.9E + 08	A	G	G	G	–	–
Chr5	2.2E + 08	–	–	Y (C)	–	A	–
Chr2	4.7E + 07	–	–	T	G	–	T
SNW-025845820.1	1379	–	–	C	–	T	–
SNW-025893768.1	4863	–	–	C	–	W (A)	–

**Note**: Y represents a pyrimidine (C) and W represents an adenine or thymine (A). “SNW” indicates scaffolds. The ‘-’ symbol indicates that the allele is heterozygous at that locus in the corresponding pepper type. This means that both the reference and alternate alleles are present in the genotype.

### 3.2 Population genetic diversity

A genetic diversity analysis was conducted using 8475 SNPs across 99 pepper lines adapted to Mediterranean climate conditions. The analysis revealed an average number of total alleles (Na) of 2.281 and the number of effective alleles (Ne) of 1.755, indicating a substantial genetic variability within the population (Table 4). Although each SNP locus in a diploid species is inherently bi-allelic, the calculation of the average number of alleles (Na) and effective number of alleles (Ne) in software like GenAlEx [42] is performed at the population level across multiple loci and individuals. This averaging process can sometimes yield values slightly above two. Several factors contribute to this outcome. Missing data, along with the method used to average alleles across the dataset, can lead to minor deviations above two alleles when interpreting population-level metrics [38,61]. Additionally, while our dataset was filtered to retain only bi-allelic SNPs, technical artifacts or low-frequency sequencing errors might momentarily appear as additional alleles before filtering thresholds remove them [39]. The computation of Na and Ne by GenAlEx considers variation across all individuals, so the arithmetic mean can be slightly greater than two even though no individual SNP locus truly has more than two alleles. We have confirmed that our SNP calling and filtering ensured that the retained markers are bi-allelic SNPs, and the slight increase above two should be viewed as a minor artifact of population-level averaging rather than evidence of truly multi-allelic loci.

**Table 4:** Summary of genetic diversity metrics for each pepper type.

Pepper Type	Na	Ne	I	Ho	He	FIS	Froh
Sivri	2.25	1.75	0.58	0.49	0.31	-0.34	0.12
Dolma	2.3	1.8	0.6	0.5	0.32	-0.35	0.11
Kil	2.2	1.7	0.57	0.48	0.3	-0.33	0.13
Charleston	2.35	1.78	0.61	0.51	0.32	-0.36	0.1
Kapia	2.28	1.76	0.59	0.49	0.31	-0.34	0.12
Mazamort	2.27	1.74	0.58	0.49	0.31	-0.34	0.11
Chili	2.29	1.77	0.59	0.5	0.32	-0.35	0.12
Jalapeno	2.32	1.79	0.6	0.5	0.32	-0.35	0.11
Mean	2.281	1.755	0.591	0.493	0.313	-0.345	0.115
SE	0.081	0.066	0.034	0.005	0.008	0.025	0.005

Note: Na =  Number of different alleles; Ne =  Effective number of alleles; I =  Shannon’s Information Index; Ho =  Observed heterozygosity; He =  Expected heterozygosity; FIS =  Inbreeding coefficient; Froh (runs of homozygosity)

The Shannon diversity index (I) averaged 0.591 (Table 4). The inbreeding coefficient (FIS) across the pepper population was found to be -0.345, indicating a significant excess of heterozygosity within the population. The observed heterozygosity (Ho) of 0.493 was notably higher than the expected heterozygosity (He) of 0.313, further confirming the occurrence of outbreeding or reduced levels of inbreeding. This negative FIS suggests that there is greater genetic diversity within the pepper population than would be expected under random mating, possibly due to gene flow or crossbreeding practices. The negative value of FIS reflects a deviation from Hardy-Weinberg equilibrium, indicating a departure from complete random mating and favoring heterozygotes over homozygotes in the population. In addition to FIS, the runs of homozygosity (Froh) values were calculated to provide a more refined measure of inbreeding. The Froh values across the population averaged 0.115, with individual pepper varieties displaying values ranging from 0.10 (Charleston) to 0.13 (Kil). The combination of low Froh values and the negative FIS supports the conclusion that this population is primarily outbred, with minimal signs of recent inbreeding. The excess heterozygosity, along with the negative FIS and low Froh values, suggests a healthy genetic diversity within the pepper population, likely a result of breeding strategies that maintain genetic variability through cross-pollination and the introduction of diverse genetic material.

The decay of linkage disequilibrium with physical distance between SNPs was examined using pairwise R^2^ values (S1 Fig.). As expected, R^2^ values generally declined as the physical distance between SNPs increased, indicating that SNPs in close proximity tend to have higher LD. This pattern is consistent with the expectation that recombination events break down LD over larger physical distances. A moderate degree of variability was observed in the R^2^ values at given distances, suggesting that additional factors, such as local recombination rates and population history, may influence LD patterns.

The nucleotide diversity, a measure of genetic variation, was calculated as π=0.403. This value represents the average number of nucleotide differences per site between two randomly chosen sequences. A π value of 0.403 indicates moderate genetic diversity within the population. This level of diversity suggests a relatively healthy population structure with sufficient variation for evolutionary potential, although it is also shaped by factors like population size, history, and selection pressures.

**Fig 1:**
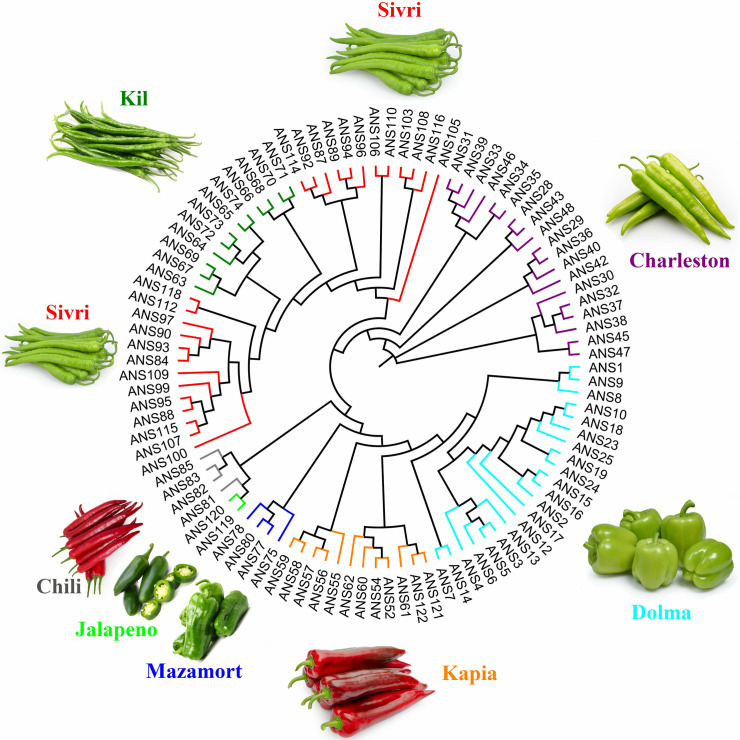
UPGMA based dendrogram generated using 8475 SNPs across 99 pepper lines.

The genetic distances between the 99 pepper lines were calculated using the distance matrix option, with values ranging from 0.314 to 0.414 (S2 Table). This range indicated a broad spectrum of genetic diversity among the lines. A phylogenetic tree was constructed using the UPGMA clustering method based on the 8475 high-quality SNPs, illustrating the genetic relationships among the pepper lines (Fig 1). The tree reveals distinct clustering patterns, with chili peppers and jalapeños forming a close genetic group, while Dolma and Charleston-type peppers exhibited more pronounced genetic divergence. The Kil-type pepper was positioned between two Sivri-type pepper lines, showing substantial genetic resemblance to both. Kapia types are placed between Dolma and Mazamort types, highlighting their intermediate genetic position.

**Fig 2:**
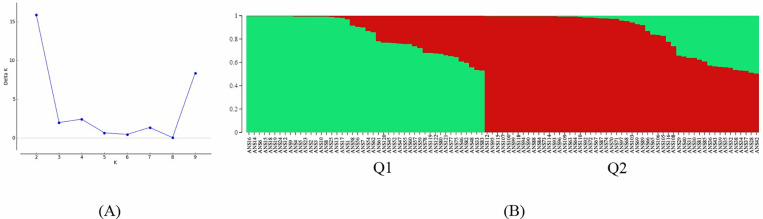
SNP marker based population structure. (A) Delta K values for different numbers of populations assumed (K) in the STRUCTURE analysis. (B) Classification of 99 pepper lines into two populations (K =  2) using STRUCTURE v2.3.4. Each accession is represented by a single row, which is partitioned into colored segments in proportion to the estimated membership in the two subpopulations. Numbers on the y-axis show the subgroup membership, and the x-axis shows the different accession.

The hierarchical population structure was analyzed, with the number of subpopulations (K) set from 2 to 10 and ten runs performed for each K-value. The optimal K value was determined using Structure Harvester, with the largest delta K observed at K =  2, suggesting the presence of two main clusters (Q1 and Q2) in the pepper panel (Fig 2A). The pepper lines were considered part of a cluster when the probability of membership threshold was above 0.50. Consequently, Q1 and Q2 contained 46 and 53 lines, respectively (S3 Table). The resulted plot showed distinct patterns of genetic clustering. Mazamort, Jalapeño, and Dolma-type peppers exclusively belong to Q1, indicating a unique genetic composition. Kapia and Charleston-type peppers predominantly fall within Q1, except for one Charleston and one Kapia-type pepper, suggesting some genetic overlap with Q2. Three chili-type peppers were categorized under Q2, while two are assigned to Q1, reflecting their genetic diversity. Q2 encompasses all Kil and Sivri-type peppers, indicating a distinct genetic group (Fig 2B).

The STRUCTURE analysis divided the 99 pepper lines into two main genetic clusters (Q1 and Q2). To assess the relationship between genetic clustering and botanical classification, we examined the distribution of pepper types within each cluster (Table 5).

**Table 5:** Distribution of pepper types within each genetic cluster identified by STRUCTURE analysis.

Pepper Type	Number of Lines	Cluster Q1	Cluster Q2
Dolma	21	21	0
Charleston	19	18	1
Kapia	12	12	0
Kil	12	0	12
Mazamort	4	4	0
Chili	5	2	3
Sivri	25	0	25
Jalapeño	2	2	0
**Total**	**99**	**59**	**40**

Cluster Q1 Predominantly included Dolma, Kapia, Charleston, Mazamort, and some Chili and Jalapeño lines. These types were generally characterized by larger, blocky or tapered fruits and are often sweet. Cluster Q1 represents sweet pepper types commonly used in Mediterranean cuisine for fresh consumption or stuffing. Cluster Q2 comprises all Sivri and Kil lines, as well as some Chili lines. These types have long, thin fruits and range from mild to very hot. Cluster Q2 represents hot or pungent pepper types used as spices or for their heat. The grouping also reflects the geographical origins, with Cluster Q1 including lines that are more widespread in the Mediterranean basin, while Cluster Q2 includes traditional Turkish varieties like Sivri and Kil.

**Fig 3:**
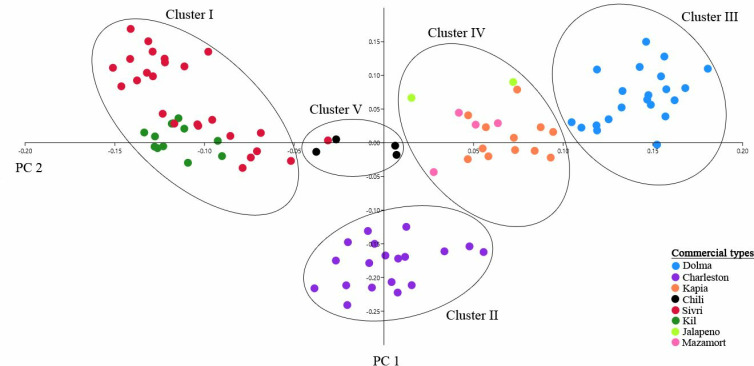
Principal coordinate analysis (PCoA) plot showing genetic relationships among 99 pepper lines. Note: The first two principal coordinates explain 10% and 15% of the total genetic variation, respectively.

The principal coordinate analysis (PCoA) identified five distinct genetic clusters among the pepper varieties, reflecting clear population structure (Fig 3). Cluster I comprised the Sivri and Kil types, showing overlap and suggesting shared genetic backgrounds or gene flow. Cluster II was distinct and included only the Jalapeño type, highlighting significant genetic differentiation. Cluster III consisted of the Dolma type, forming a tight and genetically similar group. Cluster IV included both Charleston and Kapia types, which exhibited some overlap, indicating possible gene flow or shared ancestry. Finally, Cluster V represented the Chili type, a smaller but genetically distinct group. These results underscore the genetic differentiation among the pepper varieties, with Jalapeño emerging as the most genetically distinct, while Charleston and Kapia displayed a closer genetic relationship.

## 4. Discussion

### 4.1 Efficiency and applicability of ddRADSeq in pepper genomic studies

In the current study, the ddRADSeq method was employed to generate SNPs for genetic diversity and population structure analysis in 99 pepper lines adapted to Mediterranean climate conditions. The sequencing platform produced approximately 318 million raw sequence reads, with individual lines yielding between 0.76 and 5.21 million reads. This variability in read number per line may be attributed to factors such as short read length, depth of coverage, PCR issues, and sequencing errors [62].

The pepper genome, approximately 3.5 Gb in size, is one of the largest in the Solanaceae family and is highly complex due to the prevalence of repetitive elements, which account for 75-80% of the genome [14,63]. The high number of scaffolds in the assembly likely contributes to the large number of SNPs obtained, as fragmented assemblies can artificially inflate SNP counts by creating more opportunities for variant detection within scaffolds [63]. The reference genome “UCD10Xv1.1” used in this study contains a large number of scaffolds (>81,000), comprising 16.8% of the total assembled genome [19]. Although the large number of SNPs within scaffolds can present challenges for accurately pinpointing novel gene locations in genetic mapping and genome-wide association studies (GWAS), they remain useful for comparative studies aimed at assessing genetic diversity and characterizing collections [19]. Similar findings have been observed in other studies involving large, complex genomes, such as melon and pear, where scaffold-rich assemblies also contributed to a high number of SNPs but posed challenges in gene mapping efforts [64,65]. However, the utility of these SNPs in diversity assessments and genotype comparisons remains robust, making them valuable for studies focused on population structure and germplasm characterization [66]. However, it is noteworthy that the SNP density obtained in this study differed from those in other studies, possibly due to differences in coverage values and bioinformatics approaches. Similar patterns of uneven SNP distribution have been observed in other studies of pepper genomes. For instance, Tripodi et al. [67] reported clusters of SNPs in certain genomic regions, which they attributed to variations in recombination rates and selective pressures. Additionally, the uneven distribution of SNPs observed by Tripodi et al. [67] in their characterized pepper collection could be attributed to the repetitive structure of the pepper genome, variations in selection pressure [68], and mutations [69]. The identification of high SNP density regions on certain chromosomes, such as Chromosome 1, can be targeted for MAS [70]. For example, if these regions are associated with disease resistance genes, breeders can use SNP markers within these regions to select resistant lines [71].

The presence of private alleles in specific pepper types may result from several factors, including local adaptation to environmental conditions, artificial selection during breeding, or genetic drift in isolated populations [72]. For example, unique alleles in Kil and Mazamort types could be adaptations to specific climatic conditions in their regions of cultivation or could have arisen due to selection for particular agronomic traits by breeders [41]. The identification of private alleles among different pepper types provides valuable insights into the genetic differentiation and uniqueness within the population. These private alleles, specific to certain pepper types, are indicative of unique genetic variations that can be crucial for breeding programs aiming to introduce or enhance specific traits [73]. Our discovery of private alleles unique to certain pepper types, such as the allele on Chromosome 1 found only in Charleston, Chili, Kil, and Jalapeño types, presents an opportunity to develop molecular markers for traits like disease resistance or stress tolerance.

The comprehensive genetic diversity and population structure analysis of 99 pepper lines adapted to Mediterranean climate conditions using 8475 SNPs revealed a relatively high level of diversity compared to previous studies on other cultivated pepper populations [69,74]. This high diversity provides critical insights for future pepper breeding programs, as it suggests greater genetic variability that can be harnessed to develop improved varieties. The substantial genetic variability observed, indicated by the average number of total alleles (Na =  2.281) and effective alleles (Ne =  1.755), coupled with a moderate Shannon diversity index (I =  0.591), is relatively high compared to other cultivated pepper populations [75], which often show lower diversity due to narrower breeding pools and domestication bottlenecks. This comparison underscores the rich genetic resource available within this pepper population, offering valuable potential for future breeding efforts [41].

The high observed heterozygosity (Ho =  0.493) compared to the expected heterozygosity (He =  0.313), and the negative inbreeding coefficient (FIS =  -0.345), further support the presence of a healthy genetic exchange and reduced inbreeding, making these lines particularly valuable for breeding programs [76]. Genetic distance calculations, ranging from 0.314 to 0.414, indicate a wide range of genetic diversity, which is vital for breeding programs focused on improving various traits in pepper varieties [77]. The phylogenetic tree illustrates distinct genetic relationships among the pepper lines, revealing clear clustering patterns [78]. The close genetic grouping of chili peppers and jalapeños, along with the pronounced divergence of Dolma and Charleston-type peppers, provides valuable information for breeders. For instance, the substantial genetic resemblance of Kil-type peppers to both Sivri-type lines suggests potential for cross-breeding to combine desirable traits from both types. Similarly, the intermediate position of Kapia types between Dolma and Mazamort types highlights their potential as bridges in breeding programs targeting specific traits from these groups. The STRUCTURE analysis, identifying two main genetic clusters (Q1 and Q2), offers a clear view of the underlying genetic architecture and ancestral differences within the population. The distinct clustering of Mazamort, Jalapeño, and Dolma-type peppers in Q1 indicates their unique genetic composition, which could be targeted for breeding programs focusing on specific traits. The predominant inclusion of Kapia and Charleston types in Q1, with minor overlaps in Q2, suggests a shared genetic background that could be harnessed for developing new varieties. The distinct genetic group of all Kil and Sivri-type peppers in Q2 further provides a focused target for breeding programs aimed at enhancing traits specific to these types. The genetic clusters identified through STRUCTURE analysis showed a clear correlation with the botanical classification and morphological traits of the pepper lines [67]. Cluster Q1 predominantly included sweet pepper types (*C. annuum* var. *grossum*), such as Dolma, Kapia, and Mazamort, which are characterized by larger fruits suitable for stuffing or fresh consumption. Cluster Q2 mainly consists of hot pepper types (*C. annuum* var. *longum*), including Sivri and Kil, known for their long, thin, and often pungent fruits. This separation aligns with the traditional uses and consumer preferences in the Mediterranean region [79]. The genetic differentiation between clusters suggests that traits like fruit shape, size, and pungency have a genetic basis that can be targeted in breeding programs [69]. Understanding the genetic relationships enables breeders to design crosses that combine desirable traits from different clusters [80], such as developing new varieties with the sweetness of Dolma peppers and the heat tolerance of Sivri types. The clustering reflects the domestication and selection history of peppers in the Mediterranean region, where human preferences have shaped the genetic diversity [41]. The differentiation may also indicate adaptation to specific environmental conditions, with certain types thriving in particular regions [81]. The identification of two distinct genetic clusters corresponding to sweet and hot pepper types offers practical guidance for breeders. For example, crossing sweet types from Cluster Q1 (e.g., Dolma and Kapia) with hot types from Cluster Q2 (e.g., Sivri and Kil) could combine desirable traits such as sweetness and heat tolerance, leading to new varieties that cater to diverse consumer preferences. The genetic clusters identified in our study also correspond closely with the botanical classifications of sweet and hot pepper types. This pattern is consistent with previous research indicating that fruit morphology and pungency are significant factors in pepper genetic differentiation [82].

The PCoA analysis, identifying five distinct genetic clusters (Clusters I-V), offered a more nuanced view of the underlying genetic architecture and population structure compared to the two main genetic clusters previously identified through STRUCTURE analysis. The distinct clustering of Mazamort, Jalapeño, and Dolma-type peppers in Clusters I and III indicates their unique genetic composition, which could be targeted for breeding programs focusing on specific traits such as fruit size and shape [27]. The predominant grouping of Kapia and Charleston in Cluster IV, with minimal overlap with other clusters, suggests a shared genetic background that could be harnessed for developing new varieties with combined traits from these varieties [82]. The clear separation of all Kil and Sivri-type peppers in Cluster II provides a focused target for breeding programs aimed at enhancing traits such as heat tolerance and fruit morphology, which are specific to these types. This clustering pattern aligns well with the botanical classification of the pepper lines, with sweet pepper types (e.g., Dolma, Kapia, and Mazamort) generally found in Clusters I, III, and IV [78]. In contrast, hot pepper types (e.g., Sivri and Kil) form a distinct group in Cluster II, suggesting strong genetic differentiation driven by traits like pungency and fruit shape. This separation also reflects traditional uses and consumer preferences in the Mediterranean region, where sweet peppers (C. annuum var. grossum) such as Dolma are prized for their larger fruits, and hot peppers (C. annuum var. longum) such as Sivri and Kil are favored for their pungency and elongated fruit shapes [27]. The genetic differentiation observed between these clusters suggests that traits such as fruit size, shape, and pungency have a strong genetic basis, making them ideal targets for breeding programs [13]. Understanding these genetic relationships enables breeders to design informed crosses that combine desirable traits from different clusters, such as creating new varieties that blend the sweetness of Dolma with the heat tolerance of Sivri [83]. The clustering patterns revealed in the PCoA also reflect the domestication and selection history of peppers in the Mediterranean region, where human preferences have shaped genetic diversity. The differentiation observed may further indicate adaptation to specific environmental conditions, with certain varieties thriving in particular regions. For breeders, these distinct genetic clusters offer practical guidance for designing crosses that combine advantageous traits from different groups. For instance, crossing sweet types from Cluster I (e.g., Dolma and Kapia) with hot types from Cluster II (e.g., Sivri and Kil) could produce new varieties that cater to a wider range of consumer preferences by incorporating both sweetness and heat tolerance.

The current study provided insights into the genetic diversity, population structure, and domestication-related genomic signatures of pepper lines adapted to Mediterranean climates, while comparing them with previous findings from Liu et al. [12] and Cao et al. [13]. Our results revealed a set of 8475 high-quality SNP markers, and the analyses performed highlighted a high level of genetic variability, evidenced by the observed heterozygosity (Ho =  0.493), negative inbreeding coefficient (FIS =  -0.345), and distinct clustering of pepper lines into two main genetic clusters (Q1 and Q2) and five groups based on Principal Coordinate Analysis (PCoA). These observations align well with, but also differ in important aspects from, those reported by Liu et al. [12] and Cao et al. [13] particularly with respect to genetic diversity, domestication processes, and population structure.

The negative FIS observed in our population indicated an excess of heterozygosity, suggesting outbreeding practices and relatively low levels of inbreeding, which is a feature of a healthy, diverse population. Our findings revealed substantially higher nucleotide diversity (π ≈  0.403) in Mediterranean-adapted pepper lines compared to the much lower levels (~0.001–0.003) reported by Liu et al. [12]. While Liu et al. [12] did not provide heterozygosity estimates for their *Capsicum* populations, the stark contrast in nucleotide diversity highlights the influence of sampling strategies, germplasm composition, and methodological choices on genetic diversity metrics. The populations examined by Liu et al. [12] may have experienced stronger genetic bottlenecks, encompassed a narrower genetic base, or undergone more intensive selection pressures, resulting in reduced genetic variation. In contrast, our panel of Mediterranean-adapted lines—derived from landraces, breeding programs, and diverse regional sources—likely retained broader genetic diversity, reflecting gene flow, varied selection histories, and less severe bottleneck events. Additionally, the ddRADSeq approach employed here targets relatively polymorphic genomic regions, which can inflate nucleotide diversity estimates compared to whole-genome sampling strategies. Differences in filtering parameters and thresholds (e.g., MAF, coverage depth) also contribute to divergent estimates. Thus, the discrepancies observed between our results and those of Liu et al. [12] underscore the context-dependency of genetic diversity estimates, shaped by the interplay of biological history, experimental design, and analytical frameworks. The high levels of heterozygosity underline the maintenance of genetic diversity during cultivation and improvement of *Capsicum* species. This is in contrast with domestication processes that often involve genetic bottlenecks, resulting in decreased genetic diversity. For instance, Cao et al. [13] noted a reduction in genetic diversity during the domestication of blocky-fruited *C. annuum* peppers, which was accompanied by a high level of linkage disequilibrium (LD), reflecting a genetic bottleneck. In our study, despite some geographic separation between pepper groups, evidence of substantial genetic diversity suggested a less intense bottleneck during domestication or subsequent breeding processes.

Liu et al. [12] demonstrated the development of a graph pan-genome to capture the genetic diversity across *Capsicum* species, including both domesticated and wild accessions. Their use of a pan-genome approach allowed for the identification of a large number of structural variations and SNPs, capturing significant population differentiation. Our study, using a conventional SNP panel, found consistent patterns of SNP variation, including an uneven distribution of SNP markers across chromosomes and a high density of SNPs in Chromosome NC_061115.1. This SNP density variation among chromosomes, similar to what Liu et al. [12] reported, reflects the influence of evolutionary forces such as recombination rates, selection, and structural constraints. However, unlike Liu et al. [12]‘s broad species-wide approach, our focus on Mediterranean-adapted lines provided more detailed insight into region-specific genetic adaptation.

Population structure and genetic clustering analyses in our study revealed two main genetic clusters, with different pepper types showing distinct genetic separation. Similarly, Liu et al. [12] reported clustering among different *Capsicum* species using both phylogenetic analysis and principal component analysis (PCA), but with a larger diversity of species and wider geographic distribution. Our results also identified private alleles, which are indicative of unique genetic characteristics within specific pepper types, emphasizing regional specialization within the Mediterranean germplasm. Cao et al. [13], in their work, described specific genomic regions and alleles associated with domestication events, including the introgression of traits such as fruit size, shape, and pungency. The private alleles identified in our study, particularly on Chromosomes 1, 2, and 5, could represent local adaptations and selection events similar to those highlighted by Cao et al. [13].

Domestication is a major theme across our study as well as those of Liu et al. [12] and Cao et al. [13]. In our research, the observed genetic diversity, clustering, and SNP frequencies hint at the dual forces of human-mediated selection and natural adaptation. Unlike the study by Cao et al. [13], which illustrated significant domestication-related genomic signals in specific fruit types and genetic bottlenecks during blocky pepper domestication, our study indicates that Mediterranean-adapted pepper lines may have undergone domestication with less severe genetic constriction. This difference might be attributed to the continued exchange of germplasm and gene flow in the Mediterranean region, as suggested by the low levels of inbreeding and high heterozygosity observed. Liu et al. [12]‘s identification of multiple independent domestication events among *Capsicum* species further supports the notion of diverse pathways leading to modern cultivated peppers, and our findings align with this notion by indicating the presence of unique alleles that likely originated from localized selection pressures.

Moreover, our study identified distinct genetic clusters that correspond to different morphological types, which is consistent with the domestication and diversification trajectories discussed by Cao et al. [13] who described how fruit characteristics such as size, shape, and pungency were influenced by selection for specific alleles, often resulting in divergent domestication paths. Our findings, particularly regarding SNP frequency differences between chromosomes and private alleles associated with specific pepper types, indicate that similar selective pressures may have shaped the genetic landscape of Mediterranean peppers, leading to diversity in fruit morphology and usage. For example, Q1 primarily contained sweet pepper types, while Q2 included hot and pungent types, reflecting consumer preference-driven domestication, similar to the selective forces highlighted by Cao et al. [13] during the evolution of blocky versus elongated fruit peppers.

## 5. Conclusion

Our study provided a comprehensive analysis of the genetic diversity and population structure of 99 pepper lines adapted to Mediterranean climates using ddRADSeq. The significant genetic variability and identification of distinct genetic clusters offer valuable resources for pepper breeding programs. By utilizing the identified SNP markers and private alleles, breeders can implement marker-assisted selection to develop improved varieties with desirable traits such as disease resistance, stress tolerance, and enhanced fruit quality. Future research should focus on associating these genetic markers with specific phenotypic traits to facilitate their practical application in breeding. Our study also provides a comprehensive SNP dataset and reveals significant genetic diversity among Mediterranean-adapted pepper lines. These findings have direct applications in pepper breeding programs, where the identified genetic markers can be used in MAS and GS to develop improved varieties with enhanced traits.

## Supporting information

S1 Fig The decay of linkage disequilibrium with physical distance between SNPs. (PNG)

S1 Table The details of the pepper lines and data of individual sequencing reads and GC content in the present study. (XLSX)

S2 Table Genetic distance matrix for 99 lines based on 8475 SNPs. (XLSX)

S3. Table Cluster (Q) membership based on STRUCTURE at K  =  2. (XLSX)
