# Supplementary figures and images for: Genomic insights into Mediterranean pepper diversity using ddRADSeq
Source: PLoS One. 2025 Mar 10;20(3):e0318105. doi: 10.1371/journal.pone.0318105 (PMC11892853; doi:10.1371/journal.pone.0318105)

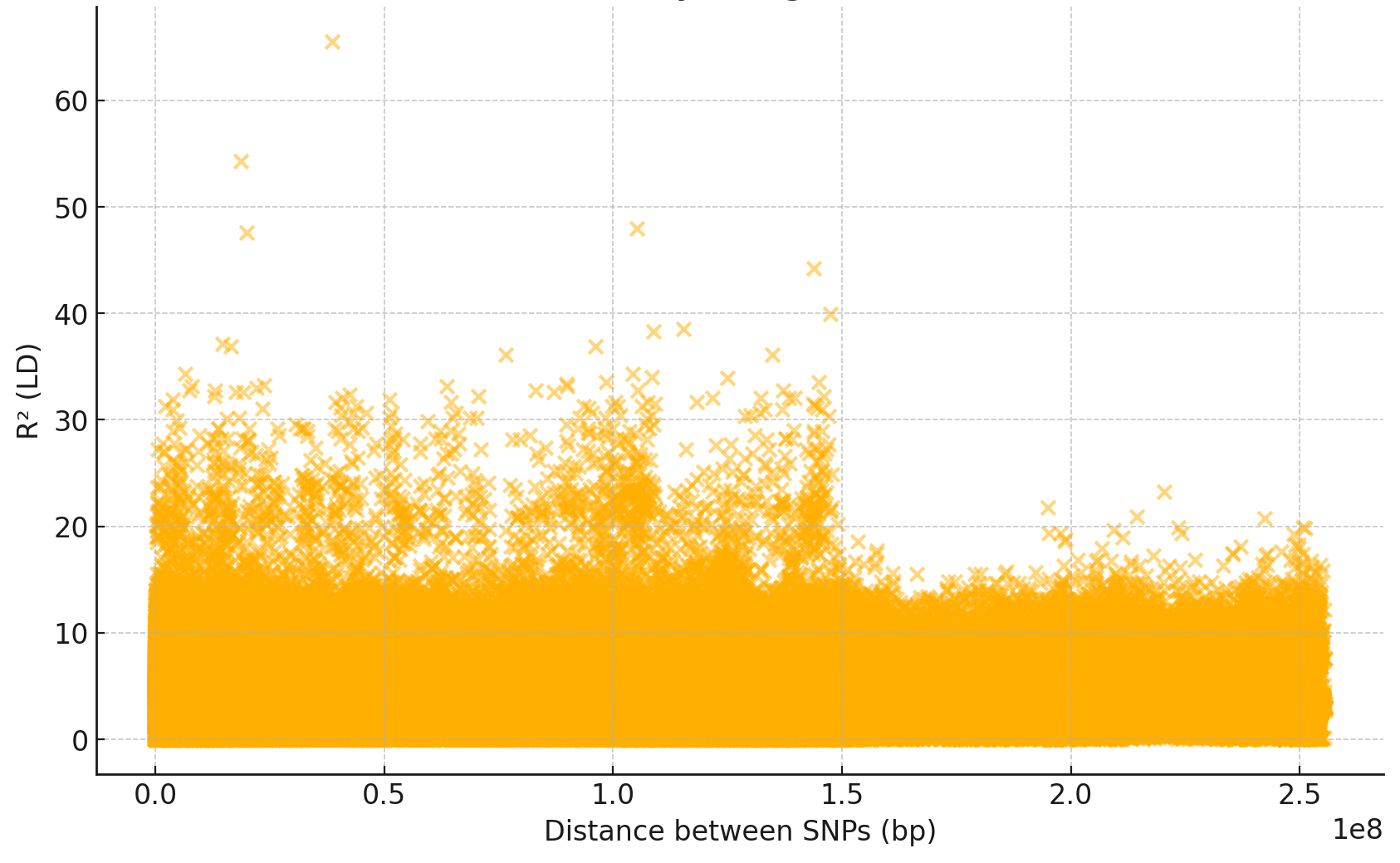

Supplement: S1 Fig — (PNG) [file pone.0318105.s001.png]
